# Estudo Observacional de Expressões Ditas por Solicitantes de Atendimento Emergencial para uma Parada Cardiorrespiratória e o Impacto no Reconhecimento pelo Médico Regulador

**DOI:** 10.36660/abc.20230343

**Published:** 2024-11-07

**Authors:** Matheus Henrique Ramos Voos, Caroline Manami Okamoto, Artur Boeck Trommer, Amanda Berlinck da Silva, Eduardo Franke da Cruz, Gustavo Andreazza Laporte, Antônio Rogério Proença Tavares Crespo, Andrea Regner, Karin Viegas

**Affiliations:** 1 Universidade Federal de Ciências da Saúde de Porto Alegre Porto Alegre RS Brasil Universidade Federal de Ciências da Saúde de Porto Alegre, Porto Alegre, RS – Brasil; 2 Porto Alegre Secretaria Municipal de Saúde Porto Alegre RS Brasil Porto Alegre Secretaria Municipal de Saúde – Assessoria de Ensino e Pesquisa,Porto Alegre, RS – Brasil; 3 Santa Casa de Misericórdia de Porto Alegre Porto Alegre RS Brasil Santa Casa de Misericórdia de Porto Alegre, Porto Alegre, RS – Brasil

**Keywords:** Parada Cardíaca Extra-Hospitalar, Assistência Pré-hospitalar, Operador de Emergência Médica

## Abstract

**Fundamento:**

A sobrevivência de uma vítima de parada cardiorrespiratória (PCR) extra-hospitalar tem relação com o fornecimento precoce de reanimação cardiopulmonar (RCP), sendo que a agilidade no reconhecimento desse evento por um médico regulador (MR) pode contribuir para a cadeia de reanimação. Atualmente, existe escassa literatura sobre o tema.

**Objetivos:**

Analisar categorias e subcategorias de palavras/expressões utilizadas espontaneamente por leigos nos chamados por PCR e sua relação com o reconhecimento desse evento pelos MR.

**Métodos:**

Estudo transversal, com análise de chamadas de socorro feitas por leigos, devido à PCR, em um serviço de atendimento móvel de urgência do Brasil. As expressões utilizadas foram classificadas em seis categorias e 31 subcategorias. A análise estatística empregou testes com modelos uni e multivariados para avaliação da força da associação das categorias e subcategorias das palavras/expressões com a presunção de PCR. O nível de significância estatística adotado foi de 5%.

**Resultados:**

Foram incluídos 284 chamados no estudo e, após aplicados os critérios de exclusão, 101 foram analisados. As categorias de expressões “estado cardiovascular/perfusional” (p=0,019) e “estado geral” (p=0,011) foram identificadas como fatores de confusão no reconhecimento de uma PCR. Ainda, as subcategorias: “dificuldade respiratória” (p=0,023), “irresponsividade verbal” (p=0,034), “cor facial” (p=0,068) e “passando mal” (p=0,013) também foram identificadas como fatores de confusão. Por outro lado, as subcategorias de expressões: “ausência de respiração” (p=0,010); “posição espacial” (p=0,016); e “emergências cardiovasculares” (p=0,045) foram identificadas como fatores facilitadores para o reconhecimento de PCR.

**Conclusão:**

Categorias e subcategorias de expressões empregadas pelos solicitantes durante o chamado por PCR podem influenciar no ágil reconhecimento dessa condição pelo médico regulador.

## Graphical abstract

**Figura Central:**
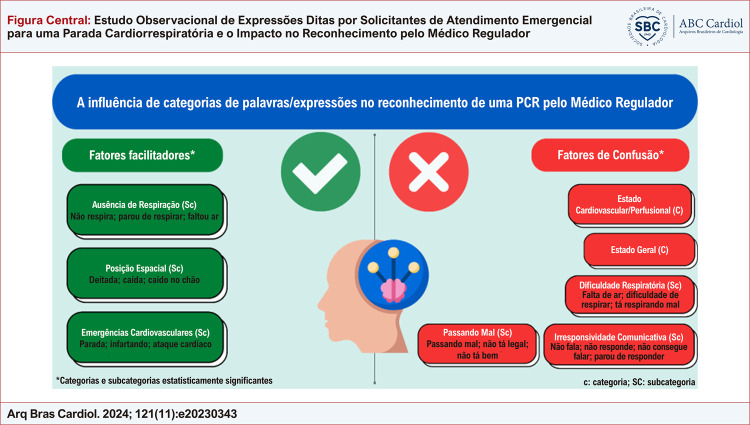
: Estudo Observacional de Expressões Ditas por Solicitantes de Atendimento Emergencial para uma Parada Cardiorrespiratória e o Impacto no Reconhecimento pelo Médico Regulador

## Introdução

O aumento da taxa de sobrevivência de uma vítima de parada cardiorrespiratória (PCR) em um ambiente extra-hospitalar depende da precocidade e qualidade da reanimação cardiopulmonar (RCP).^1^ Visto que a maioria das PCRs ocorre fora do ambiente hospitalar,^2^ fica evidente que o reconhecimento rápido desse agravo é o pilar da cadeia de sobrevivência.^3^

No Brasil, a assistência pré-hospitalar pública é realizada pelo Serviço de Atendimento Móvel de Urgência (SAMU) que, além de enviar uma equipe para realizar o atendimento no local, instrui ao solicitante medidas que devem ser realizadas até a chegada do socorro. Esse componente do atendimento é realizado pelo médico regulador (MR) – profissional responsável por reconhecer rapidamente a PCR e orientar ao solicitante a realização de RCP de forma assistida.^4^ O reconhecimento de uma PCR por um profissional de atendimento emergencial aumenta a taxa de sobrevivência da vítima e diminui o tempo até o fornecimento de RCP.^5,6^

Apesar da importância da realização precoce da RCP, mais da metade das ligações para serviços de emergência devido a PCR não são seguidas pela realização de RCP por parte dos indivíduos presentes na cena.^7,8^ Alguns fatores que contribuem para a demora ou a falta do reconhecimento da PCR incluem: descrição superficial da respiração; perguntas desnecessárias; e problemas técnicos e humanos na comunicação.^9-11^Segundo o *International Liaison Committee on Resuscitation (ILCOR),* porém, existe uma lacuna na literatura científica sobre aspectos que influenciam a cadeia de tomada de decisões pelo MR.^12^ Por exemplo, a associação entre as categorias de palavras/expressões chaves relacionadas com uma PCR usadas pelos solicitantes durante o chamado e o efetivo reconhecimento pelo MR sobre a real ocorrência de PCR.

Logo, o presente estudo tem por objetivo: (i) descrever as categorias e subcategorias de palavras/expressões empregadas pelos solicitantes leigos no chamado por PCR e (ii) analisar a associação entre as categorias e subcategorias de palavras/expressões e o reconhecimento pelo MR de PCR ocorrida no local do chamado.

## Métodos

O atendimento do SAMU inicia pela recepção do chamado por um técnico auxiliar de regulação médica (TARM), responsável por identificar a queixa principal do solicitante e seus dados de identificação. Se necessário, o solicitante é transferido para o atendimento com um MR, o qual deve presumir o tipo de afecção e a sua gravidade para tomada de decisão sobre as orientações para o solicitante e o envio do socorro para o suporte mais adequado. Avaliados esses fatores, o MR decide pelo envio de uma unidade básica (tripulada por um técnico em enfermagem e um motorista) e/ou de uma avançada (tripulada por um médico, um enfermeiro e um motorista).^4^

### Desenho do estudo

Estudo transversal, documental, com análise de chamados emergenciais remotos (registrados em prontuário eletrônico e áudios) de vítimas de PCR atendidas pelo SAMU em Porto Alegre/RS, Brasil.

### Amostra

A amostra foi consecutiva e não probabilística, sendo composta por todos os chamados que tiveram como socorro comprovado PCR de origem não-traumática, pela equipe de atendimento do SAMU, durante o período de 01 de março ao dia 31 de outubro de 2019.

### Critérios de Inclusão

Chamadas de socorro para o SAMU por solicitantes leigos em que foi enviado socorro e a equipe do SAMU reconheceu, na cena, PCR de origem não-traumática.

### Critérios de Exclusão

Os critérios de exclusão foram: PCR de origem traumática; a ausência de áudios com o TARM ou com o MR; ligação realizada de uma instituição de saúde; solicitante não estava na cena; o solicitante era ou houve o relato da presença de um profissional da saúde na cena; e problemas técnicos que impossibilitaram a compreensão do áudio do MR pelos pesquisadores.

### Fonte dos dados

O SAMU utiliza um sistema informatizado para registro do serviço de atendimento pré-hospitalar SAMU 192. Durante o período estudado, foram utilizados para a análise os registros digitais e áudios das versões *SAPH Cliente 2.18.3.5 e SAPH Reports 2.18.2.1.* A extração dos chamados foi realizada pela empresa responsável pelo *software*, que os selecionou e os categorizou como clínicos/PCR. Após essa seleção preliminar, foi possível definir as fichas de atendimento e os áudios a serem analisados.

Os registros digitais e áudios foram analisados pelos pesquisadores, os quais preencheram um instrumento de pesquisa. Foram extraídos dados sobre local de ocorrência, gravidade presumida pelo MR (pequena, média, alta, morte e indeterminada), gravidade comprovada pela equipe de atendimento no local, deslocamento de equipe de apoio e desfecho do atendimento (óbito ou não óbito). Esses dados foram extraídos, codificados e digitados em uma planilha no programa *Microsoft Excel 2020®*.

Para diminuir possíveis fatores de confusão, os pesquisadores realizaram um treinamento quanto à identificação dos fatores de seleção nos chamados, assim como em relação à categorização das palavras/expressões utilizadas pelos solicitantes.

### Categorias de palavras/expressões

As palavras/expressões foram classificadas em categorias e subcategorias ainda não validadas na literatura, devido à carência de estudos relacionados a esse tema. Entretanto, os artigos de Berdowski et al.^5^ e Tamminen et al.^13^ serviram como base para a construção das categorias. Algumas adaptações foram feitas para adequação de linguagem, sendo estabelecidas, então, seis categorias e 31 subcategorias, apresentadas na Tabela 1.

**Tabela 1:** – Categorias e subcategorias das palavras/expressões utilizadas por solicitantes leigos durante as chamadas por socorro para descrever pacientes com PCR

Categoria	Subcategoria	Descrição dos leigos
Estado Ventilatório	Ausência de respiração	Não respira, parou de respirar, acho que não respira, faltou o ar*
	Dificuldade respiratória	Falta de ar, dificuldade de respirar, tá respirando mal, tentando respirar*
	Respiração anormal	Roncando, engasgando, respiração esquisita*
	Frequência anormal	Respira bem pouquinho, não respira regularmente, tá parando de respirar*
	Profundidade anormal	Não sinto mais a respiração, respiração fraca, respiração curta, respiração profunda*
	Alterações visuais decorrentes de problemas ventilatórios	Não mexe nada o peito, boca aberta tentando respirar*
	Outros	Respira, tá respirando*
Estado de Consciência e Responsividade	Posição espacial	Deitado, no chão, caída, atirado, caído no chão*
	Desmaio	Desmaiada, desmaiou*
	Irresponsividade geral	Não reage, desacordada, inconsciente, apagado*
	Irresponsividade comunicativa	Não fala, não responde, não tem condições de falar, não consegue falar, parou de responder*
	Irresponsividade motora	Parado, não consegue caminhar, não consegue levantar*
	Estado confusional	Mole, tonta*
	Outros	Fechando os olhos, perdendo os sentidos, tá acordado*
Estado Cardiovascular/ Perfusional	Manifestações cardíacas	Dor no peito, palpitação, pressão tá baixando*
	Temperatura	Gelada, fria*
	Cor facial	Roxo, pálida, boca roxa, arroxeado*
	Sudorese	Suando, suando gelado, suadouro*
	Pulso	Sem pulso*
Estado Geral	Passando mal	Passando mal, não tá legal, não tá passando bem*
	Presunção de óbito	Quase morta, acho que faleceu, entrando óbito, desfalecida*
Emergência Presumida	Emergência cardiovascular	Parada, infartando, PCR, ataque cardíaco*
	Convulsão/ataque epiléptico	Ataque epiléptico, em crise, convulsão*
	Outros	Mal súbito*
Outros	Manifestações orofaríngeas	Babando, boca aberta, saindo gosma pela boca, mordendo a língua*
	Manifestações oculares	Revirando os olhos, olhos abertos*
	Manifestações em membros	Pesando os braços, afrouxou as pernas*
	Manifestações gastrointestinais	Vômito, náusea*
	Manifestações urológicas	Se mijando*
	Manifestações nasais	Sangrou pelo nariz*
	Outros	Sacudir, tremendo, barulhos*

*Palavras e expressões semanticamente similares foram consideradas adaptado de Berdowski et al.^5^ e Tamminen et al.^13^ PCR: parada cardiorrespiratória.

### Análise estatística

Os dados categóricos foram apresentados em frequência e percentual, enquanto os dados contínuos em média e desvio padrão. A comparação do socorro presumido como PCR com os atendimentos foi realizada utilizando-se os testes Qui-Quadrado e Exato de *Fisher*. Para as análises de associações do socorro presumido como PCR com as variáveis que envolviam a classificação e subclassificação das palavras/expressões foram aplicados modelos uni e multivariados de regressão logística com as estimativas de *Odds Ratio* (OR) com Intervalo de Confiança (IC) de 95%. A interpretação dos valores de OR deve ser feita da seguinte forma: os chamados em que a categoria ou subcategoria não estavam presentes, foram definidos como OR = 1 para a presunção da PCR; um OR > 1 indica que a presença daquela categoria/subcategoria aumenta a chance de o MR classificar o chamado como não-PCR (errando o diagnóstico); e um OR < 1 informa que a presença da categoria/subcategoria diminui a chance de o MR classificar o chamado como não-PCR (acertando o diagnóstico). Passaram para etapa multivariada as variáveis cujo valor foi p <0,20 na análise univariada. A análise dos dados foi realizada utilizando-se o *software SPSS* versão 25.0 e o nível de significância adotado foi de 5%.

### Aspectos éticos

O estudo foi aprovado pelo Comitê de Ética em Pesquisa da Secretaria Municipal de Saúde de Porto Alegre (CAAE #4.287.099).

## Resultados

**Figura 1:**
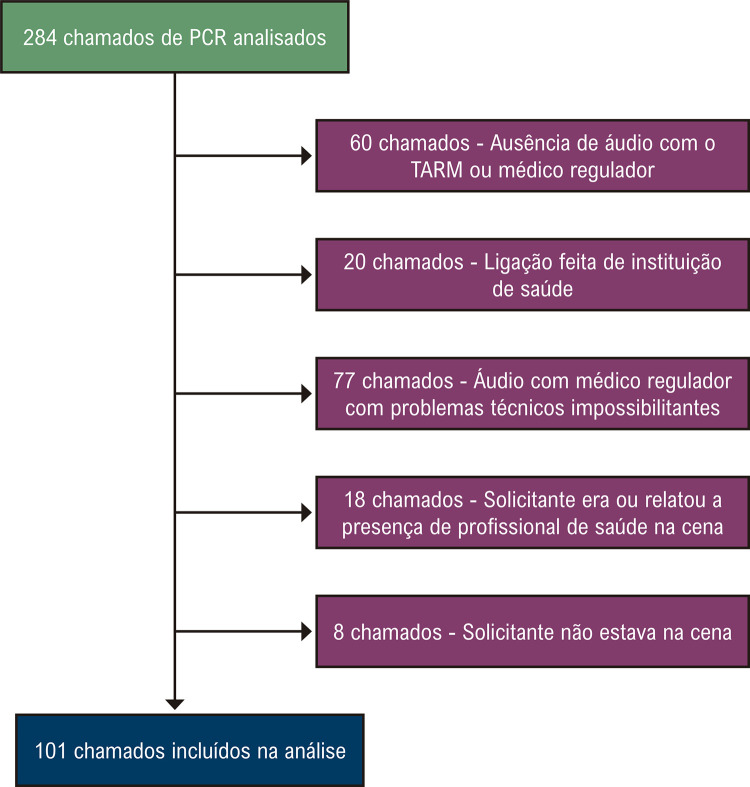
– Fluxograma representando a seleção de chamados incluídos na amostra analisada no estudo. PCR: parada cardiorrespiratória; TARM: técnico auxiliar de regulação médica.

Foram incluídos no presente estudo 284 chamados; todavia, após avaliação seguindo os critérios de seleção, 101 chamados foram analisados (Figura 1). Os dados de caracterização da amostra, conforme o tipo de socorro presumido pelo MR estão apresentados na Tabela 2. As vítimas de PCR encontravam-se majoritariamente em ambientes residenciais (82%), seguidas de locais públicos ou privados (9%), como lojas, supermercados e shoppings. Em relação aos dados sobre os atendimentos, o MR presumiu corretamente o socorro, como PCR, em 40 chamados (40%) do total da amostra analisada. No que concerne à mortalidade, foi verificado que em 81% dos atendimentos o paciente foi a óbito na cena, sendo que essa taxa foi significativamente maior quando o MR identificava a PCR.

**Tabela 2:** – Caracterização dos atendimentos dos chamados com socorro comprovado como parada cardiorrespiratória pelo SAMU em uma capital do sul do Brasil, 2019

Variáveis			Socorro Presumido PCR		p
		Amostra (n=101)	Sim (n=40)	Não (n=61)	
		n (%)	n (%)	n (%)	
Locais de ocorrência, n (%)	Residência	88 (87)	33 (83)	50 (82)	0,049^¥^
	Locais públicos ou privados*	9 (9)	6 (15)	3 (5)	
	Casas geriátricas	1 (1)	1 (3)	0 (0)	
	Via Pública^†^	1 (1)	0 (0)	1 (2)	
	Indeterminado	7 (7)	0 (0)	7 (12)	
Gravidade presumida, n (%)	Pequena	7 (7)	0 (0,0)	7 (12)	<0,001^¥^
	Média	30 (30)	1 (3)	29 (48)	
	Severa	56 (55)	39 (98)	17(28)	
	Morte	1 (1)	0 (0)	1 (2)	
	Indeterminada	7 (7)	0 (0)	7 (12)	
Gravidade confirmada, n (%)	Pequena	0 (0)	0 (0)	0 (0)	0,176^¥^
	Média	2 (2)	1 (3)	1 (2)	
	Severa	28 (28)	7 (18)	21 (34)	
	Morte	71 (70)	32 (80)	39 (64)	
	Indeterminada	0 (0)	0 (0)	0 (0)	
Deslocamento de equipe de apoio, n (%)	Sim	79 (78)	27 (68)	52 (85)	0,035^¥^
	Não	22 (22)	13 (33)	9 (15)	
Óbito, n (%)	Sim	82 (81)	37 (93)	45 (74)	0,018^∆^
	Não	19 (19)	3 (8)	16 (26)	

Fonte: Autores. *lojas, supermercados, shopping; †locais externos (rua). ¥ Teste Qui-Quadrado; ∆ Teste Exato de Fisher.

As informações completas sobre as categorias e subcategorias, conforme o socorro presumido, estão apresentadas nas Tabelas 3 (categorias) e 4 (subcategorias). As categorias de palavras/expressões mais utilizadas foram: Estado de Consciência/Responsividade (ECR) (68%), seguida de Estado Ventilatório (EV) (65%). Na análise univariada, palavras/expressões sobre Estado Geral (EG) e Estado Cardiovascular/Perfusional (ECP) foram identificadas como fatores de confusão para a decisão do MR sobre o socorro presumido. Quando realizada a análise multivariada, apenas o EG apresentou um valor significativo como fator de confusão para o MR. Na categoria emergência presumida (EP), a subcategoria “emergências cardiovasculares” foi a mais utilizada, tendo sido empregada em 18% de todas as PCR, representando um fator facilitador na decisão de socorro presumido como PCR pelo MR.

**Tabela 3:** – Associação das categorias de palavras/expressões empregadas durante as chamadas por socorro com o reconhecimento de parada cardiorrespiratória pelo médico regulador do SAMU, Brasil, 2019

Categoria	O tipo de socorro presumido pelo MR foi PCR?							
	Sim (n=40)	Não (n=61)	Univariado			Multivariado		
	n (%)	n (%)	p	OR	IC95%	p	OR	IC95%
Estado Ventilatório	26 (65)	40 (66)	0,953	1,03	0,44 - 2,37	-	-	-
Estado de Consciência/Responsividade	26 (65)	43 (71)	0,562	1,29	0,55 - 3,01	-	-	-
Estado Cardiovascular/Perfusão	9 (23)	28 (46)	0,019*	2,92	1,19 - 7,17	0,126	2,21	0,80 - 6,12
Estado Geral	6 (15)	24 (39)	0,011*	3,68	1,34 - 10,08	0,013^#^	3,87	1,33 - 11,29
Emergência Presumida	13 (33)	12 (20)	0,147	0,51	0,20 - 1,27	0,548	0,73	0,26 - 2,02
Outros	10 (25)	24 (39)	0,139	1,95	0,81 - 4,70	0,299	1,70	0,62 - 4,64

Fonte: autores; SAMU: Serviço de Atendimento Móvel de Urgência. O “n” utilizado representa os chamados em que as palavras/expressões das respectivas categorias estiveram presentes. IC: intervalo de confiança; MR: médico regulador; OR: odds ratio; PCR: parada cardiorrespiratória. * Regressão logística univariada. # Regressão logística multivariada.

**Tabela 4:** – Associação das subcategorias de palavras/expressões empregadas durante as chamadas por socorro com o reconhecimento de parada cardiorrespiratória pelo médico regulador do SAMU. Brasil. 2019

Categoria/Subcategorias	O tipo de socorro presumido foi PCR?									
	Sim (n=40)	Não (n=61)	Univariado				Multivariado			
	n (%)	n (%)	p	OR	IC95%		p	OR	IC95%	
Estado Ventilatório										
Ausência de Respiração	21 (52,5)	12 (19,7)	0,001*	0,22	0,09 - 0,54		0,010^#^	0,23	0,07 - 0,70	
Dificuldade Respiratória	3 (7,5)	17 (27,9)	0,019*	4,77	1,29 - 17,54		0,023^#^	6,99	1,31 - 37,38	
Respiração Anormal	1 (2,5)	6 (9,8)	0,188	4,25	0,49 - 36,76		0,354	3,26	0,27 - 39,70	
Frequência Anormal	0 (0,0)	7 (11,5)	-	-	-		-	-	-	
Profundidade Anormal	4 (10,0)	4 (6,6)	0,534	0,63	0,15 - 2,69		-	-	-	
Alterações Visuais Decorrentes de Problemas Ventilatórios	0 (0,0)	2 (3,3)	-	-	-		-	-	-	
Outros	0 (0,0)	4 (6,6)	-	-	-		-	-	-	
**Estado de Consciência/Responsividade**										
Posição Espacial	13 (32,5)	10 (16,4)	0,063	0,41	0,16 - 1,05		0,016^#^	0,19	0,05 - 0,73	
Desmaio	8 (20,0)	13 (21,3)	0,874	1,08	0,40 - 2,91		-	-	-	
Irresponsividade Geral	6 (15,0)	17 (27,9)	0,137	2,19	0,78 - 6,15		0,386	1,90	0,45 - 8,11	
Irresponsividade Verbal	3 (7,5)	12 (19,7)	0,105	3,02	0,79 - 11,48		0,034^#^	7,25	1,16 - 45,25	
Irresponsividade Motora	2 (5,0)	4 (6,6)	0,747	1,33	0,23 - 7,64		-	-	-	
Estado Confusional	2 (5,0)	4 (6,6)	0,747	1,33	0,23 - 7,64		-	-	-	
Outros	2 (5,0)	3 (4,9)	0,985	0,98	0,16 - 6,16		-	-	-	
**Estado Cardiovascular/Perfusão**										
Manifestações Cardíacas	0 (0,0)	3 (4,9)	-	-	-		-	-	-	
Temperatura	2 (5,0)	5 (8,2)	0,540	1,70	0,31 - 9,20		-	-	-	
Cor	7 (17,5)	21 (34,4)	0,068	2,47	0,94 - 6,54		0,128	2,85	0,74 - 11,00	
Sudorese	0 (0,0)	3 (4,9)	-	-	-		-	-	-	
Pulso	1 (2,5)	2 (3,3)	0,822	1,32	0,12 - 15,08		-	-	-	
**Estado Geral**										
Passando Mal	4 (10,0)	22 (36,1)	0,006*	5,08	1,60 – 16,16		0,013^#^	6,75	1,50 – 30,35	
Presunção de Óbito	3 (7,5)	3 (4,9)	0,594	0,64	0,12 – 3,33		-	-	-	
**Emergência Presumida**										
Emergências Cardiovasculares	11 (27,5)	7 (11,5)	0,045*	0,34	0,12 - 0,98		0,210	0,42	0,11 - 1,62	
Convulsão/Ataque Epiléptico	2 (5,0)	4 (6,6)	0,747	1,33	0,23 - 7,64		-	-	-	
Outros	0 (0,0)	1 (1,6)	-	-	-		-	-	-	
**Outros**										
Manifestações Orofaríngeas	9 (22,5)	11 (18,0)	0,582	0,76	0,28 - 2,04		-	-	-	
Manifestações Oculares	0 (0,0)	6 (9,8)	-	-	-		-	-	-	
Manifestações Membros	0 (0,0)	2 (3,3)	-	-	-		-	-	-	
Náuseas/Vômitos	1 (2,5)	3 (4,9)	0,550	2,02	0,20 - 20,z11		-	-	-	
Manifestações Urológicas	0 (0,0)	1 (1,6)		-	-		-	-	-	
Manifestações Nasais	0 (0,0)	1 (1,6)	-	-	-		-	-	-	
Outros	0 (0,0)	3 (4,9)	-	-	-		-	-	-	

Fonte: autores SAMU: Serviço de Atendimento Móvel de Urgência. O “n” utilizado representa os chamados em que as palavras/expressões das respectivas subcategorias estiveram presentes. IC: intervalo de confiança; MR: médico regulador; OR: odds ratio PCR: parada cardiorrespiratória; * Regressão logística univariada. ^#^ Regressão logística multivariada.

No que se refere à categoria ECR, as subcategorias mais utilizadas foram: “posição espacial” (23%) e “irresponsividade geral” (23%). A “posição espacial” foi um fator facilitador significativo na avaliação correta do socorro presumido como PCR pelo MR. Por outro lado, a outra subcategoria da ECR “irresponsividade verbal” representou um fator de confusão significativo para a tomada de decisão do MR na definição do socorro presumido.

Dentre as subcategorias da categoria EV, a mais utilizada foi “ausência de respiração”, sendo usada em 32% de todos os chamados de PCR. Essa subcategoria de expressão apresentou valor significativo como fator facilitador para a presunção correta da PCR, tanto na análise univariada, quanto na multivariada. Porém, foi observado que a subcategoria “dificuldade respiratória” foi um fator de confusão significativo para presunção de PCR pelo MR. Da mesma forma, na categoria EG, a subcategoria mais empregada foi “passando mal” (26%), a qual, também, representou um fator de confusão para a definição do socorro presumido como PCR. A subcategoria mais utilizada na categoria ECP foi “cor facial” (28%). No que concerne à categoria “Outros” (OT), a subcategoria mais utilizada foi “manifestações orofaríngeas” (20%). Nenhuma das subcategorias da ECP ou da OT teve associação com a presunção de PCR pelo MR. Interessante que seis por cento da amostra de solicitantes mencionou algum sinal que poderia estar relacionado a uma convulsão tônico-clônica da vítima durante o chamado por PCR.

## Discussão

No presente estudo, a partir da análise de 101 chamados, definimos categorias e subcategorias das expressões mais utilizadas por solicitantes leigos, nos pedidos de socorro por PCR. Identificamos associações entre algumas categorias e subcategorias das expressões usadas no chamado com o reconhecimento da PCR pelo médico regulador. Este é um estudo precursor no país que contribui para o debate científico mundial sobre aspectos que influenciam a cadeia de tomada de decisões pelo MR. Em 2018, a ILCOR indicou que estudos que avaliassem as lacunas de conhecimento em relação ao reconhecimento da PCR extra-hospitalar deveriam ser considerados de elevado impacto e prioridade.^12^ Verificamos que a presunção adequada de PCR pelo MR (40% dos chamados) foi abaixo da média descrita em estudos prévios sobre o tema. Uma revisão sistemática,^14^ realizada em 2015, incluindo 16 estudos que analisaram 6955 chamados, relatou sensibilidade global de reconhecimento de PCR, por médicos reguladores, de 74%. Entretanto, nessa revisão, identificou-se grande heterogeneidade dos resultados, havendo acentuada variação de sensibilidade (entre 14% e 97%).^14^ Corroborando os achados dessa revisão, outros estudos mostraram amplo espectro de sensibilidade (37-96%) dos MR no reconhecimento de PCR em chamados por leigos.^13,15,16^ Essa variabilidade pode ser explicada pelas diferenças significativas encontradas na forma como os MR aderem aos protocolos de cada local, além dos diversos fatores sociais, culturais e financeiros relacionados a cada sistema de emergência analisado.^17,18^

Em relação às categorias de palavras/expressões mais utilizadas durante o chamado – ECR e EV – nosso estudo está alinhado a estudos realizados em outros países. Em um estudo na Inglaterra, o termo “inconsciente” somado a um ou mais dos sintomas “não respirando”, “respiração ineficaz” e “respiração barulhenta” foi utilizado em 80% de todos os chamados por PCR.^15^ Além disso, em uma revisão sistemática de 23 estudos, incluindo quatro que analisaram diretamente os áudios dos chamados de PCR, uma combinação de “inconsciência” e “ausência de respiração” ou “presença de respiração anormal” foram os termos mais comumente relatados.^19^ Segundo o *European Resuscitation Council*, o reconhecimento de uma PCR, em ambiente extra-hospitalar, deve ser baseado na combinação do paciente estar inconsciente e apneico ou respirando de forma anormal.^20^ De fato, dentre as subcategorias da categoria EV, a mais utilizada (“ausência de respiração”), teve efeito facilitador para o reconhecimento da PCR pelo MR.

A nossa análise da subcategoria “dificuldade respiratória” como fator de confusão para o reconhecimento da PCR também corresponde às evidências atuais. Apesar dos estudos reconhecerem a importância de identificação da respiração anormal, uma razão comumente indicada para a não identificação da PCR é a má interpretação ou falta de clareza em relação ao estado de respiração.^12,17,18,21^ Na Holanda, um estudo mostrou que se atingiria uma sensibilidade de 100% caso todos os chamados analisados contendo os termos “respiração anormal” ou “ausente” fossem classificados como PCR; porém, essa medida de regulação poderia causar altíssima taxa de falso-positivos (80%).^5^ Ainda, em um estudo realizado, na Noruega, concluiu-se que a respiração anormal continua a ser a principal barreira para o reconhecimento da parada cardíaca.^14^ Frente às evidências, destacamos a importância de se perguntar, ao solicitante, sobre a qualidade da respiração da vítima, caso a primeira resposta tenha sido inconclusiva. Interessante, ainda, destacar que, na categoria ECR, verificamos que a “posição espacial” foi um fator facilitador para a assertividade da decisão do médico como evento de PCR, enquanto a “irresponsividade verbal” foi um fator de confusão. Reforçando esse achado, palavras-chave como “desabou” ou “caído” estão entre as mais usadas para se descrever PCR.^13^

Em relação a outros fatores de confusão, identificamos mais duas categorias de palavras/expressões: EG e ECP. De fato, expressões como “pulso anormal” e “frequência cardíaca anormal” diminuíram a probabilidade de uma chamada de PCR ser corretamente identificada pelo MR.^15^ Essas evidências corroboram que o foco de indagação do MR, em suspeita de PCR, deve ser o estado de consciência e o EV, uma vez que a percepção do leigo a respeito da presença/alteração de pulso poderia retardar o fornecimento de RCP e o envio de suporte médico.^20,22^ O fato de que nenhuma das subcategorias da categoria ECP ter influenciado no reconhecimento da PCR aponta para o baixo valor discriminativo desses sinais e sintomas, principalmente em um país onde a população tem heterogeneidade em relação ao nível de instrução formal.^23^ Estudos identificaram que as palavras como “o paciente está azul” estavam presentes em 18% dos chamados confirmados de PCR,^13^ enquanto expressões relacionadas à mudança de cor corresponderam a aproximadamente 29% dos chamados.^5,15^ A descrição da vítima como “roxo, cinza e branco” foi significativa para o reconhecimento de uma PCR por um serviço de emergência remoto.^5^ A expressão mais utilizada na categoria EG foi a subcategoria “passando mal” (26%), a qual representou um fator de confusão para a presunção de PCR pelo MR. Em contraste ao nosso achado, outro estudo identificou que a palavra-chave “passando mal” (*unwell*) foi utilizada em menor proporção (18%) dos chamados por PCR confirmadas.^5^ Na categoria EP, a subcategoria “emergências cardiovasculares” foi a mais utilizada (18%), sendo um fator facilitador na decisão do tipo de socorro presumido pelo MR. Nesse sentido, estudo prévio demonstrou que a expressão “ataque cardíaco” esteve presente em 11% dos chamados por PCR e “parada cardíaca” em 4%, sendo ambas expressões facilitadoras do reconhecimento de PCR.^5^ Embora nenhuma subcategoria da OT tenha tido influência significativa no reconhecimento da PCR pelo MR, destaca-se que diversos termos dessas subcategorias podem ser confundidos com outros quadros clínicos prevalentes (ex. quadros convulsivos - “revirando os olhos”, “se babando”, “sacudindo”). Porém, é fato que em um curto período de movimentos similares, as convulsões podem ocorrer durante a isquemia encefálica global no início de uma PCR.^20^

À luz do conjunto de evidências, percebemos que são diversos os fatores, durante o chamado por socorro, que podem interferir na assertividade de reconhecimento da PCR pelo MR. Entre estes, destacam-se: o nível de instrução do solicitante, questões de comunicação e falta de programas, em grande escala, para formação de primeiros respondentes.^9,24^ Além disso, demonstram a necessidade de definição de protocolos de reconhecimento de PCR e programas de treinamento para as equipes de médicos reguladores. Verificamos que as equipes de apoio, no SAMU, foram deslocadas para atendimento no local em 78% dos 101 chamados. Interessante observar que houve aumento significativo de envio de ambulâncias quando o MR não identificava a PCR durante o chamado. Quando a equipe de atendimento básico chegava à cena e identificava que era uma PCR, era necessário o envio de uma segunda equipe de suporte avançado para realizar o atendimento adequado. Parece contraditório o aumento significativo da mortalidade das vítimas quando o MR identificava a PCR durante o chamado de socorro, uma vez que a expectativa seria o oposto dado o atendimento feito mais precocemente.^5,6^ Todavia, possivelmente, a identificação da PCR pelo MR durante o chamado por socorro, tenha correspondido à maior probabilidade de morte da vítima do que quando o paciente apresentava manifestações inconclusivas ou precoces da PCR. Outra questão a ser considerada seria a janela de tempo entre o evento da PCR e o chamado do solicitante, parâmetro que não pôde ser aferido por falta dessa informação.

### Relevância clínica e perspectivas futuras

Este é o primeiro estudo, no Brasil, que analisa áudios de chamadas por socorro por PCR, avaliando categorias e subcategorias de palavras/expressões que influenciam no reconhecimento desse agravo pelo MR do SAMU. Esse estudo fomenta a realização de investigações futuras no tema para qualificar o processo de regulação médica dos chamados por emergências clínicas, particularmente da PCR. Combinações de palavras/expressões chave poderão ser utilizadas para a implementação de protocolos visando a qualificação da identificação precoce da PCR pelo MR. Os resultados também sugerem a necessidade de melhorar o processo de comunicação entre leigos e médicos reguladores, para aumentar a assertividade do reconhecimento e, portanto, precocidade da RCP na PCR. Já existem sistemas de reconhecimento automático de fala que demonstraram melhor assertividade do que de médicos reguladores.^25^ Logo, uma associação de termos empregados pelos solicitantes, por meio de reconhecimento automático de fala associado a *machine learning,* poderá trazer uma perspectiva de resposta em grande escala para chamados por emergências médicas.

### Limitações

O presente estudo foi piloto de um modelo de análise que ainda tem muitos fatores a serem ajustados e melhorados. Dentre as limitações do estudo, devemos considerar a falta ou inconsistência de registros e as condições ruins de gravação e armazenamento de alguns chamados, ocasionando perda de casos da amostra inicial. Ademais, não avaliamos a variabilidade do conhecimento e experiência entre os médicos reguladores, nem seu grau de treinamento e de adesão aos protocolos de regulação. Também não avaliamos as competências de comunicação, com público leigo, entre os médicos reguladores. Outrossim, precisamos considerar a grande variação do nível de instrução e de conhecimento dos primeiros respondentes e dos solicitantes. Por fim, não existem registros do intervalo de tempo entre o colapso da vítima e a ligação por socorro do solicitante.

Como limitantes contextuais ao presente estudo devemos considerar o próprio funcionamento da assistência pré-hospitalar pública no Brasil - que, ao não se utilizar de protocolos de envio de socorro mais efetivos, nem utilizar reconhecimento automatizado das ligações recebidas, contribui para informações altamente dependentes da interpretação e análise pessoal de cada médico regulador. Nesse sentido, outro fator limitante relacionado à comunicação interpessoal podem ser as diversidades linguísticas regionais, podendo a tradução de palavras/expressões não refletir as singularidades culturais e educacionais locorregionais.

## Conclusões

As palavras/expressões consideradas nas categorias ECP e EG e nas subcategorias ”dificuldade respiratória”, “irresponsividade verbal”, “cor facial” e “passando mal” foram identificadas como fatores de confusão para a identificação da PCR pelo MR. Por outro lado, as subcategorias “ausência de respiração”, “posição espacial” e “emergências cardiovasculares” foram identificadas como fatores facilitadores da presunção de PCR pelo MR. O conhecimento sobre os fatores de confusão para o reconhecimento da PCR pode auxiliar na produção de protocolos assistenciais e de estratégias de treinamento mais efetivos para o MR, qualificando a comunicação com o solicitante leigo. Em contraste, as expressões facilitadoras poderão ser rotineiramente incorporadas aos protocolos de regulação de casos suspeitos de PCR. Todavia, mais estudos nessa área são necessários devido à ampla variedade de fatores que influenciam o efetivo processo de comunicação entre usuário e médico regulador nos cenários extra-hospitalares de atendimento à PCR.

## References

[1] Hasselqvist-Ax I, Riva G, Herlitz J, Rosenqvist M, Hollenberg J, Nordberg P (2015). Early Cardiopulmonary Resuscitation in Out-of-hospital Cardiac Arrest. N Engl J Med.

[2] Zheng ZJ, Croft JB, Giles WH, Mensah GA (2001). Sudden Cardiac Death in the United States, 1989 to 1998. Circulation.

[3] Panchal AR, Bartos JA, Cabañas JG, Donnino MW, Drennan IR, Hirsch KG (2020). Part 3: Adult Basic and Advanced Life Support: 2020 American Heart Association Guidelines for Cardiopulmonary Resuscitation and Emergency Cardiovascular Care. Circulation.

[4] Ciconet RM (2015). Response Time of the Brazilian Mobile Emergency Servisse.

[5] Berdowski J, Beekhuis F, Zwinderman AH, Tijssen JG, Koster RW (2009). Importance of the First Link: Description and Recognition of an Out-of-hospital Cardiac Arrest in an Emergency Call. Circulation.

[6] Kuisma M, Boyd J, Väyrynen T, Repo J, Nousila-Wiik M, Holmström P (2005). Emergency Call Processing and Survival from Out-of-hospital Ventricular Fibrillation. Resuscitation.

[7] Nichol G, Thomas E, Callaway CW, Hedges J, Powell JL, Aufderheide TP (2008). Regional Variation in Out-of-hospital Cardiac Arrest Incidence and Outcome. JAMA.

[8] Vaillancourt C, Stiell IG, Canadian Cardiovascular Outcomes Research Team (2004). Cardiac Arrest Care and Emergency Medical Services in Canada. Can J Cardiol.

[9] Michiels C, Clinckaert C, Wauters L, Dewolf P (2021). Phone CPR and Barriers Affecting Life-saving Seconds. Acta Clin Belg.

[10] Bång A, Herlitz J, Holmberg S (2000). Possibilities of Implementing Dispatcher-assisted Cardiopulmonary Resuscitation in the Community. An Evaluation of 99 Consecutive Out-of-hospital Cardiac Arrests. Resuscitation.

[11] Eisenberg MS (2006). Incidence and Significance of Gasping or Agonal Respirations in Cardiac Arrest Patients. Curr Opin Crit Care.

[12] Olasveengen TM, Caen AR, Mancini ME, Maconochie IK, Aickin R, Atkins DL (2017). 2017 International Consensus on Cardiopulmonary Resuscitation and Emergency Cardiovascular Care Science with Treatment Recommendations Summary. Resuscitation.

[13] Tamminen J, Lydén E, Kurki J, Huhtala H, Kämäräinen A, Hoppu S (2020). Spontaneous Trigger Words Associated with Confirmed Out-of-hospital Cardiac Arrest: A Descriptive Pilot Study of Emergency Calls. Scand J Trauma Resusc Emerg Med.

[14] Viereck S, Møller TP, Rothman JP, Folke F, Lippert FK (2017). Recognition of Out-of-hospital Cardiac Arrest During Emergency Calls - A Systematic Review of Observational Studies. Scand J Trauma Resusc Emerg Med.

[15] Watkins CL, Jones SP, Hurley MA, Benedetto V, Price CI, Sutton CJ (2021). Predictors of Recognition of Out of Hospital Cardiac Arrest by Emergency Medical Services Call Handlers in England: A Mixed Methods Diagnostic Accuracy Study. Scand J Trauma Resusc Emerg Med.

[16] Lu CH, Fang PH, Lin CH (2019). Dispatcher-assisted Cardiopulmonary Resuscitation for Traumatic Patients with Out-of-hospital Cardiac Arrest. Scand J Trauma Resusc Emerg Med.

[17] Kirby K, Voss S, Bird E, Benger J (2021). Features of Emergency Medical System Calls that Facilitate or Inhibit Emergency Medical Dispatcher Recognition that a Patient is in, or at Imminent Risk of, Cardiac Arrest: A Systematic Mixed Studies Review. Resusc Plus.

[18] Kirby K, Voss S, Benger J (2023). Identifying Patients at Imminent Risk of Out-of-hospital Cardiac Arrest During the Emergency Medical Call: The Views of Call-takers. Resusc Plus.

[19] Vaillancourt C, Charette ML, Bohm K, Dunford J, Castrén M (2011). In Out-of-hospital Cardiac Arrest Patients, does the Description of Any Specific Symptoms to the Emergency Medical Dispatcher Improve the Accuracy of the Diagnosis of Cardiac Arrest: A Systematic Review of the Literature. Resuscitation.

[20] Perkins GD, Graesner JT, Semeraro F, Olasveengen T, Soar J, Lott C (2021). European Resuscitation Council Guidelines 2021: Executive Summary. Resuscitation.

[21] Hardeland C, Sunde K, Ramsdal H, Hebbert SR, Soilammi L, Westmark F (2016). Factors Impacting Upon Timely and Adequate Allocation of Prehospital Medical Assistance and Resources to Cardiac Arrest Patients. Resuscitation.

[22] Berg KM, Cheng A, Panchal AR, Topjian AA, Aziz K, Bhanji F (2020). Part 7: Systems of Care: 2020 American Heart Association Guidelines for Cardiopulmonary Resuscitation and Emergency Cardiovascular Care. Circulation.

[23] Al Hasan D, Yaseen A, El Sayed M (2020). Epidemiology and Outcomes from Out-of-hospital Cardiac Arrest in Kuwait. Emerg Med Int.

[24] Potts J, Lynch B (2006). The American Heart Association CPR Anytime Program: The Potential Impact of Highly Accessible Training in Cardiopulmonary Resuscitation. J Cardiopulm Rehabil.

[25] Blomberg SN, Folke F, Ersbøll AK, Christensen HC, Torp-Pedersen C, Sayre MR (2019). Machine Learning as a Supportive Tool to Recognize Cardiac Arrest in Emergency Calls. Resuscitation.

