# Supplementary material for: Estudo Observacional de Expressões Ditas por Solicitantes de Atendimento Emergencial para uma Parada Cardiorrespiratória e o Impacto no Reconhecimento pelo Médico Regulador
Source: Arq Bras Cardiol. 2024 Nov 7;121(11):e20230343. [Article in Portuguese] doi: 10.36660/abc.20230343 (PMC11634301; doi:10.36660/abc.20230343)
Supplement: Translation

# Translation: Observational Study of Words Used by Emergency Callers and Their Impact on the Recognition of an Out-Of-Hospital Cardiopulmonary Arrest by the Medical Dispatcher

**DOI:** 10.36660/abc.20230343i

**Authors:** Matheus Henrique Ramos Voos, Caroline Manami Okamoto, Artur Boeck Trommer, Amanda Berlinck da Silva, Eduardo Franke da Cruz, Gustavo Andreazza Laporte, Antônio Rogério Proença Tavares Crespo, Andrea Regner, Karin Viegas

**Affiliations:** 1 Universidade Federal de Ciências da Saúde de Porto Alegre Porto Alegre RS Brazil Universidade Federal de Ciências da Saúde de Porto Alegre, Porto Alegre, RS – Brazil; 2 Porto Alegre Secretaria Municipal de Saúde Porto Alegre RS Brazil Porto Alegre Secretaria Municipal de Saúde – Assessoria de Ensino e Pesquisa, Porto Alegre, RS – Brazil; 3 Santa Casa de Misericórdia de Porto Alegre Porto Alegre RS Brazil Santa Casa de Misericórdia de Porto Alegre, Porto Alegre, RS – Brazil

**Keywords:** Out-of-Hospital Cardiac Arrest, Prehospital Care, Emergency Medical Dispatcher

## Abstract

**Background:**

Survival of victims of out-of-hospital cardiopulmonary arrest (CA) is related to the time and quality of cardiopulmonary resuscitation (CPR).^1^ Considering that most CAs occur outside the hospital setting, it is evident that the early recognition of this condition is the cornerstone of the chain of survival. Current literature on the theme is still scarce.

**Objectives:**

To analyze categories and subcategories of words and expressions spontaneously used by laypeople during emergency calls for CA and their relationship with the recognition of this event by the medical dispatcher.

**Methods:**

This was a cross-sectional study, with analysis of calls made by laypeople due to suspected CA to emergency medical services in Brazil. The expressions used were classified into six categories and 31 subcategories. Univariate and multivariate models were used to assess the strength of the association of categories and subcategories of words and expressions with the presumption of CA. The level of significance was set at 5%. Results: A total of 284 calls were included, and after applying the inclusion criteria, 101 calls were analyzed. The categories “cardiovascular/perfusion status” (p=0.019) and “general status” (p=0.011) were identified as confounding factors for the recognition of CA, and the subcategories “breathing difficulty” (p=0.023), “verbal unresponsiveness” (p=0.034), “facial coloration” (p=0.068) and “feeling unwell” (p=0.013) were also considered as confounders. On the other hand, the subcategories “not breathing” (p=0.010); “spatial position” (p=0.016), and “cardiovascular emergencies” (p=0.045) were identified as facilitating factors for the recognition of CA.

**Conclusion:**

Categories and subcategories of expressions used by emergency callers for CA can influence the timely recognition of this condition by the medical dispatcher.

## Introduction

The increase in survival rates of patients with out-of-hospital cardiopulmonary arrest (CA) depends on the timing and quality of cardiopulmonary resuscitation (CPR).^1^ Considering that most CAs occurs outside the hospital setting,^2^ it is undeniable that the early recognition of this condition is the cornerstone of the chain of survival.^3^

In Brazilian public health system, pre-hospital care is provided by emergency medical services (SAMU, *Serviço de Atendimento Móvel de Urgência* in Portuguese), which, in addition to send a specialized personnel staff to provide local support, gives proper instructions to the bystander until help arrives at the scene. This component of care is made by a medical dispatcher (MD), who is responsible for promptly identifying a CA and assisting the caller to perform CPR.^4^The identification of a CA by an emergency provider increases survival rates and reduces the time to CPR.^5,6^ Despite the importance of an early CPR, more than half of the emergency calls due to CA are not followed by CPR performed on the scene.^7,8^Factors that contribute to the delay or lack of CPR identification include a superficial description of breathing, irrelevant questioning, and both technical and human communication issues.^9-11^

However, according to the International Liaison Committee on Resuscitation (ILCOR), a gap in the scientific literature exists regarding aspects that influence the decision-making framework by the MD.^12^ For example, the association of key words/expressions related to a CA used by the caller during the emergency call and the effective recognition of CA by the MD.

Therefore, the present study aims (i) to describe the categories and subcategories of words/expressions used by the layperson during a CA call; and (ii) to analyze the association of categories and subcategories of words/expressions with the recognition of a CA by the MD.

## Methods

The emergency service (SAMU) starts with the call response by a call handler, who is responsible for identifying the reason for the call and the caller’s identification. If it is deemed necessary, the caller is transferred to a MD, who should recognize the condition and its severity to make decisions on the instructions to be given to the caller and activates the appropriate support services. Subsequently, the MD decides which ambulance services are appropriate for the case, either basic support services (an ambulance with a nurse technician and a driver) or advanced support services (an ambulance with a MD, a nurse and a driver).^4^

### Study design

This was a cross-sectional, documental study of analysis of emergency calls (recorded in electronic medical records or audio recordings) of CA victims treated by SAMU in Porto Alegre, Brazil.

### Sample

We studied a consecutive, non-probabilistic sample composed of emergency calls, which resulted in the support for proven nontraumatic CA by the SAMU from March 01 to October31, 2019.

### Inclusion criteria

Emergency calls made by laypeople that resulted in the dispatch of support services and the recognition, by the SAMU, of a CA of nontraumatic origin on the scene.

### Exclusion criteria

The exclusion criteria were traumatic CA; absence of recordings of conversations with the call handler or the MD; telephone calls made by a healthcare center; the caller was not present on the scene; the caller was a healthcare professional or there was a health professional on the scene; and technical problems that affected the understanding of the audio recordings of the MD by the researchers.

### Data source

SAMU uses a computerized system for the registration of the pre-hospital service ordered through SAMU192. During the study period, analysis was made of the digital records and audio recordings in the versions *SAPH Cliente 2.18.3.5* and *SAPH Reports 2.18.2.1.* The software company extracted and selected the calls and categorized them as “clinical/CA”. Following this preliminary selection, the evaluation forms and the audio recordings for analysis were selected.

The digital registries and audios were examined by the researchers, who filled out a specific form. Data on the CA scene location, event severity (low, medium, high, death, unknown), severity confirmed by SAMU on the scene, dispatch of the support team and result of the care provided (death or not). These data were extracted, encoded and typed into a Microsoft Excel 2020 spreadsheet.

To minimize potential cofounders, the researchers conducted a training to identity selection factors in the calls and to categorize the words/expressions used by the callers.

### Categories of words/expressions

The words/expressions were classified into categories and subcategories, which have not been validated in the literature yet due to the scarcity of studies on the theme. However, the studies by Berdowski et al.^5^ and Tamminen et al.^13^ served as a basis for the construction of the categories. Adaptations were made for language adequacy. Six categories and 31 subcategories were generated (Table 1).

**Table 1:** – Categories and subcategories of words / expressions used to describe cardiopulmonary arrest victims by laypeople in emergency calls

Categories	Subcategories	Description by laypeople
Ventilation status	Absent breathing	Not breathing, stopped breathing, I think he/she is not breathing, out of breath*
	Breathing difficulty	Shortness of breath, breathing difficulty, he/she is not breathing well, he/she is trying to breath
	Abnormal breathing	Snoring, gasping, weird breathing*
	Abnormal rate	Breathing too slowly, not breathing regularly, stopping breathing*
	Abnormal depth	Can’t feel his/her breathing, weak breathing, short breathing, deep breathing*
	Vision problems due to ventilation problems	The chest is not moving, mouth open trying to breath*
	Others	Breathing, he/she is breathing*
Consciousness and responsiveness	Spatial position	Lying on the floor, fallen down, collapsed, fallen on the floor*
	Syncope	Fainted, passed out*
	General unresponsiveness	Does not react, unconscious, knocked out*
	Communication unresponsiveness	Does not speak, does not respond, cannot speak, stopped responding*
	Motor unresponsiveness	Cannot move, walk or stand up*
	Confusional state	Weak, dizzy*
	Others	Eyes are closing, passing out, he/she is awake*
Cardiovascular/ Perfusion status	Clinical manifestations	Chest pain, palpitations, pressure is falling*
	Temperature	Very cold, cold*
	Facial coloration	Purple, pale, purple lips, purplish*
	Sweating	Sweating, cold sweating, sweating profusely*
	Pulse	Without a pulse*
General status	Feeling unwell	Feeling unwell, not “ok”, not feeling well*
	Presumption of death	Almost dead, I think he/she passed away, dying, passed out*
Presumption of emergency	Cardiovascular emergency	Cardiac arrest, infarcted, CA, heart attack*
	Seizure/ epileptic attack	Epileptic attack, in a crisis, seizure*
	Others	Collapse*
Others	Oropharyngeal manifestations	Drooling, open mouth, froth coming out of his/her mouth, biting his/her tongue*
	Ocular manifestations	Rolling his/her eyes, eyes are open*
	Limb manifestations	Arms are heavy, relaxed his/her legs*
	Gastrointestinal manifestations	Vomit, nausea*
	Urological manifestations	He/she is pissing*
	Nasal manifestations	His/her nose is bleeding*
	Others	Shake, shivering, noises*

*Semantically similar words and expressions were also considered; adapted from Berdowski et al.^5^ e Tamminen et al.^13^. CA: cardiopulmonary arrest.

### Statistical analysis

Categorical data were expressed as frequency and percentage, and continuous data as mean and standard deviation. Comparisons of the emergency service dispatched assuming a CA with the care actually provided were made by chi-squared test and Fisher’s exact test. Analysis of the associations between the service dispatched assuming a CA and the variables involving the classification and subclassification of words/expressions was made by univariate and multivariate logistic regression models, with odds ratio (OR) estimates and 95% confidence interval (95%CI). OR should be interpreted as follows: OR=1 when the category or the subcategory of the calls was not present for CA presumption; OR>1 when the presence of that category or subcategory increases the chance of the MD classifying the call as a non-CA (misdiagnosing); and an OR <1 indicates that the presence of the category or subcategory reduces the chance of the MD classifying the call as a non-CA (making a correct diagnosis). Variables with a p<0.20 in the univariate analysis were introduced into the multivariate analysis. The analyses were made using the SPSS software version 25.0 and the level of significance was set at 5%.

### Ethical issues

The study was approved by the ethics committee of the Secretary of Health of Porto Alegre (CAAE # 4.287.099).

## Results

A total of 284 were included in the study, and after applying the inclusion criteria, 101 calls were analyzed (Figure 1).

**Figure 1:** – Flowchart of selection of the calls included in the sample analyzed in the study. CA: cardiopulmonary arrest.

Characteristics of the sample, by type of the support presumed by the MD are described in Table 2. Most of CAs occurred at home (82%), followed by public or private locations (9%), like stores, supermarkets and malls. Regarding the type of service dispatched in response to the call, the MD made a correct judgement, including the recognition of a CA, in 40 (40%) of the calls analyzed. With respect to mortality, 81% of the patients that received emergency care died at the scene, and this rate was significantly higher when the MD recognized a CA.

**Table 2:** – Characteristics of SAMU emergency response to calls for confirmed cardiopulmonary arrest in a capital in the south of Brazil, 2019

Variables			Presumption of CA		p
		Sample (n=101)	Yes (n=40)	No (n=61)	
		n (%)	n (%)	n (%)	
Scene location, n (%)	Home	88 (87)	33 (83)	50 (82)	0.049^¥^
	Public or private places*	9 (9)	6 (15)	3 (5)	
	Homes for the Aged	1 (1)	1 (3)	0 (0)	
	Public access places^†^	1 (1)	0 (0)	1 (2)	
	Indetermined	7 (7)	0 (0)	7 (12)	
Presumption of severity, n (%)	Low	7 (7)	0 (0,0)	7 (12)	<0.001^¥^
	Medium	30 (30)	1 (3)	29 (48)	
	High	56 (55)	39 (98)	17(28)	
	Death	1 (1)	0 (0)	1 (2)	
	Indetermined	7 (7)	0 (0)	7 (12)	
Confirmation of severity, n (%)	Low	0 (0)	0 (0)	0 (0)	0.176^¥^
	Medium	2 (2)	1 (3)	1 (2)	
	High	28 (28)	7 (18)	21 (34)	
	Death	71 (70)	32 (80)	39 (64)	
	Indetermined	0 (0)	0 (0)	0 (0)	
Dispatch of a support team, n (%)	Yes	79 (78)	27 (68)	52 (85)	0.035^¥^
	No	22 (22)	13 (33)	9 (15)	
Death, n (%)	Yes	82 (81)	37 (93)	45 (74)	0.018^∆^
	No	19 (19)	3 (8)	16 (26)	

SAMU: emergency medical services (Serviço de Atendimento Móvel de Urgência in Portuguese). *stores, supermarkets, mall; †outdoor locations (street); CA: cardiopulmonary arrest. ¥ chi-squared test; ∆ Fisher’s exact test.

All data on the categories and subcategories by type of service dispatched (presuming or not a CA) are described in Table 3 (categories) and Table 4 (subcategories). The most used categories of words/expressions were level of consciousness/responsiveness (LCR) (68%), followed by ventilation status (VS) (65%). In the univariate analysis, the words/expressions about general status (GS) and cardiovascular/perfusion status (CPS) were identified as cofounders for the decision making by the MD about the type of service to be dispatched to the scene. In the multivariate analysis, only GS showed a significant value as a confounder for the decision making by the MD. In “presumed emergency” (PE), the subcategory “cardiovascular emergencies” was the most used, in 18% of all CAs, representing a facilitating factor in the dispatcher’s decision for a CA.

**Table 3:** – Association of categories of words/expressions used during emergency calls with recognition of cardiopulmonary arrest by the SAMU medical dispatcher, Brazil, 2019

Categories	Presumption of CA?							
	Yes (n=40)	No (n=61)	Univariate			Multivariate		
	n (%)	n (%)	p	OR	95%CI	p	OR	95%CI
Ventilation status	26 (65)	40 (66)	0.953	1.03	0.44 - 2.37	-	-	-
Level of consciousness/responsiveness	26 (65)	43 (71)	0.562	1.29	0.55 - 3.01	-	-	-
Cardiovascular/Perfusion status	9 (23)	28 (46)	0.019*	2.92	1.19 - 7.17	0.126	2.21	0.80 - 6.12
General status	6 (15)	24 (39)	0.011*	3.68	1.34 - 10.08	0.013^#^	3.87	1.33 - 11.29
Presumption of emergency	13 (33)	12 (20)	0.147	0.51	0.20 - 1.27	0.548	0.73	0.26 - 2.02
Others	10 (25)	24 (39)	0.139	1.95	0.81 - 4.70	0.299	1.70	0.62 - 4.64

“n” represents the number of calls in which the words and expressions of respective categories/expressions were present. CI: confidence interval; OR: odds ratio; CA: cardiopulmonary arrest. * Univariate logistic regression. ^#^ Multivariate logistic regression

**Table 4:** – Association of subcategories of words/expressions used during emergency calls with recognition of cardiopulmonary arrest by the SAMU medical dispatcher, Brazil, 2019

Categories/Subcategories	Presumption of CA?									
	Yes (n=40)	No (n=61)	Univariate				Multivariate			
	n (%)	n (%)	p	OR	95%CI		p	OR	95%CI	
Ventilation Status										
Not breathing	21 (52.5)	12 (19.7)	0.001*	0.22	0.09 - 0.54		0.010^#^	0.23	0.07 - 0.70	
Breathing difficulty	3 (7.5)	17 (27.9)	0.019*	4.77	1.29 - 17.54		0.023^#^	6.99	1.31 - 37.38	
Abnormal breathing	1 (2.5)	6 (9.8)	0.188	4.25	0.49 - 36.76		0.354	3.26	0.27 - 39.70	
Abnormal rate	0 (0.0)	7 (11.5)	-	-	-		-	-	-	
Abnormal depth	4 (10.0)	4 (6.6)	0.534	0.63	0.15 - 2.69		-	-	-	
Visual changes due to ventilation problems	0 (0.0)	2 (3.3)	-	-	-		-	-	-	
Others	0 (0.0)	4 (6.6)	-	-	-		-	-	-	
**Consciousness/Resposiveness**										
Spatial position	13 (32.5)	10 (16.4)	0.063	0.41	0.16 - 1.05		0.016^#^	0.19	0.05 - 0.73	
Faint	8 (20.0)	13 (21.3)	0.874	1.08	0.40 - 2.91		-	-	-	
General unresponsiveness	6 (15.0)	17 (27.9)	0.137	2.19	0.78 - 6.15		0.386	1.90	0.45 - 8.11	
Verbal unresponsiveness	3 (7.5)	12 (19.7)	0.105	3.02	0.79 - 11.48		0.034^#^	7.25	1.16 - 45.25	
Motor unresponsiveness	2 (5.0)	4 (6.6)	0.747	1.33	0.23 - 7.64		-	-	-	
Confusional state	2 (5.0)	4 (6.6)	0.747	1.33	0.23 - 7.64		-	-	-	
Others	2 (5.0)	3 (4.9)	0.985	0.98	0.16 - 6.16		-	-	-	
**Cardiovascular/Perfusion Status**										
Cardiac manifestations	0 (0.0)	3 (4.9)	-	-	-		-	-	-	
Temperature	2 (5.0)	5 (8.2)	0.540	1.70	0.31 - 9.20		-	-	-	
Color	7 (17.5)	21 (34.4)	0.068	2.47	0.94 - 6.54		0.128	2.85	0.74 - 11.00	
Sweating	0 (0.0)	3 (4.9)	-	-	-		-	-	-	
Pulse	1 (2.5)	2 (3.3)	0.822	1.32	0.12 - 15.08		-	-	-	
**General Status**										
Feeling unwell	4 (10.0)	22 (36.1)	0.006*	5.08	1.60 – 16.16		0.013^#^	6.75	1.50 – 30.35	
Presumption of death	3 (7.5)	3 (4.9)	0.594	0.64	0.12 – 3.33		-	-	-	
**Presumption of Emergency**										
Cardiovascular emergencies	11 (27.5)	7 (11.5)	0.045*	0.34	0.12 - 0.98		0.210	0.42	0.11 - 1.62	
Seizure/Epileptic attack	2 (5.0)	4 (6.6)	0.747	1.33	0.23 - 7.64		-	-	-	
Others	0 (0.0)	1 (1.6)	-	-	-		-	-	-	
**Others**										
Oropharyngeal manifestations	9 (22.5)	11 (18.0)	0.582	0.76	0.28 - 2.04		-	-	-	
Ocular manifestations	0 (0.0)	6 (9.8)	-	-	-		-	-	-	
Limb manifestations	0 (0.0)	2 (3.3)	-	-	-		-	-	-	
Nausea/vomiting	1 (2.5)	3 (4.9)	0.550	2.02	0.20 - 20.11		-	-	-	
Urological manifestations	0 (0.0)	1 (1.6)		-	-		-	-	-	
Nasal manifestations	0 (0.0)	1 (1.6)	-	-	-		-	-	-	
Others	0 (0.0)	3 (4.9)	-	-	-		-	-	-	

“n” represents the number of calls in which the words and expressions of respective subcategories were present. CI: confidence interval; OR: odds ratio; CA: cardiopulmonary arrest; *Univariate logistic regression. ^#^ Multivariate logistic regression.

For the LCR category, the most used subcategories were “spatial position” (23%) and “general unresponsiveness” (23%). “Spatial position” was a significant facilitating factor for the correct judgement of a CA by the MD. On the other hand, the other subcategory of the LCR category – “verbal unresponsiveness” represented a significant confounder for the decision-making by the MD in the definition of the type of care to be dispatched.

Regarding the VS category, the most used subcategories were “absent breathing”, used by 32% of the calls for CA. This subcategory showed a significant value as a facilitating factor for the correct assumption of a CA, both in the univariate and the multivariate analysis. However, “breathing difficulty” was a significant confounder for the recognition of CA by the MD. Likely, in the GS category, the most used subcategory was “feeling unwell” (26%), which also represented a confounder factor for the definition of care (CA or not). In the CPS category, the most used subcategory was “facial coloration” (28%). In the “others” (OT) category, the most used subcategory “oropharyngeal manifestations” (20%). None of the subcategories of the CPS or the OT categories was associated with the presumption of a CA by the MD. Interestingly, in our sample, 6% of the callers mentioned a sign that could be related with tonic-clonic seizures of the victim during the call for CA.

## Discussion

In the present study, from the analysis of 101 calls, we defined categories and subcategories of the most used expressions by laypeople during calls for CAs. We found associations between some categories and subcategories of the expressions used during the calls in which a CA was recognized by the MD (medical dispatcher).

This is a pioneer study in Brazil that contributes to the international scientific debate about aspects that influence the decision-making framework by the MD. In 2018, the ILCOR reported that studies evaluating knowledge gaps on the recognition of out-of-hospital CA are of high impact and priority.^12^ The correct CA recognition by the MD (40% of the calls) was lower than that reported in previous studies. A systematic review^14^ conducted in 2015 included 16 studies that analyzed 6,955 calls and reported a global recognition of CA by medical dispatchers of 74%. However, this study reported a high heterogeneity of results, with high sensitivity variation (14%-97%).^14^ Indeed, other studies also reported high variation of sensitivity (37-96%) in the recognition of CA by medical dispatchers in emergency calls made by laypeople.^13,15,16^ This variability may be explained by differences in adherence to local protocols by the dispatchers, in addition to social, cultural and financial factors related to each emergency center.^17,18^

Regarding the most used words/expressions during the calls – LCR and VS – our study is in agreement with results of previous studies conducted in other countries. In a study conducted in England, the term “unconscious” plus one or more of the symptoms “not breathing”, “ineffective breathing”, and “noisy breathing” was used in 80% of all calls for CA.^15^ Besides, in a systematic review of 23 studies, including four studies that analyzed the audios of the emergency calls, a combination of “unconscious” and “not breathing” or “abnormal breathing” were the most commonly used terms.^19^ According to the European Resuscitation Council, for the recognition of an out-of-hospital CA, the patient should be unconscious and apneic or without normal breathing.^20^ In fact, in the VS category, the most commonly used subcategory – “absent breathing” – had a facilitating effect on the CA recognition by the MD.

Our analysis of the subcategory “breathing difficulty” as a confounder for the recognition of CA is also in line with current evidence. Although studies have recognized the importance of identifying abnormal breathing, one commonly mentioned reason for non-recognition of a CA is misinterpretation or lack of clarity regarding breathing status.^12,17,18,21^ A Dutch study showed that a 100% sensitivity would be achieved if all calls containing the terms “abnormal breathing” or “absent breathing” were classified as CA. However, this measure would result in a high false-positive rate (80%).^5^ In addition, the authors of a study conducted in Norway concluded that “abnormal breathing” is the main barrier to the recognition of a cardiac arrest.^14^ This body of evidence highlights the importance of asking the caller about the breathing status of the victim, in case the first response is inconclusive. Also, it is interesting to mention that, in the LCR category, “spatial position” was a facilitator to the MD to make a correct recognition of CA, whereas “verbal unresponsiveness” was a confounding factor. Words like “collapsed” or “fallen down” are among the most frequently words to describe CA.^13^

With respect to other confounding factors, two other categories of words/expressions were identified in the present study – GS and CPS. In fact, expressions like “abnormal pulse” and “abnormal heart rate” reduced the likelihood of a correct recognition of a CA call by the MD.^15^ This evidence corroborates the fact that the focus of the questions asked by the MD should be consciousness and VS, since the recognition of abnormalities in the pulse or heart rate by a layperson could delay CPR and dispatch of medical support.^20,22^ The fact that none of the subcategories of the PCS category have influenced the recognition of a CA indicates a low discriminative power of these signs and symptoms, especially in a country with a heterogeneous population regarding formal education.^23^ Studies have found that words like “the patient is blue” were present in 18% of the calls in which a CA was confirmed,^13^ whereas expressions related to change of facial coloration corresponded to nearly 29% of the calls.^5,15^ The description of the victim as “purple, grey o white” color was important for the recognition of a CA by a remote emergency service.^5^The most used words in the GS category was the “feeling unwell” subcategory (26%), which represented a confounder for the recognition of a CA by the MD. In contrast, another study reported that the word “unwell” was used in a smaller proportion (18%) of the calls for confirmed CA, and was a facilitator in the decision making on the type of emergency service to be dispatched (basic or advanced). In this respect, a previous study demonstrated that the words “heart attack” were present in 11% of the CA calls and “cardiac arrest” in 4%, and both expressions were facilitators of CA recognition.^5^ Although none of the OT subcategories had a significant influence on the recognition of the CA by the MD, it is of note that other subcategories may be confounded by other prevalent clinical conditions (e.g. seizures – “rolling eyes”, “drooling”, “shaking”). However, in a short period of similar movements, seizures may occur during global cerebral ischemia in the onset of CA.^20^

In light of this evidence, we conclude that several factors related to an emergency call may interfere with the correct recognition of CA by the MD. These include the caller’s education level, communication issues, and lack of a highly accessible training in CPR for call handlers.^9,24^ Besides, these factors demonstrate the need to establish protocols for CA recognition and training programs for MDs. Medical emergency teams were dispatched to the call scene in 78% of the total of 101 calls. Interestingly, there was a significant increase in the dispatch of ambulances when the MD did not recognize the CA during the call. When the staff of basic life support arrived at the scene and identified a CA, the staff of advanced life support was dispatched to the scene to provide appropriate care. It seems contradictory the significant increase in mortality of the victims when the medical dispatcher recognized the CA, considering the provision of earlier care.^5,6^ However, it is possible that the recognition of the CA by the MD during the emergency call corresponded to higher likelihood of death than in case of individuals with early or inconclusive manifestations of CA. Another aspect to be considered is the time span between the CA and the call made by the victim or bystander; this information was not available and could not be analyzed.

### Clinical relevance and future perspectives

This is the first study in Brazil to analyze audio recordings of out-of-hospital CA calls for categories and subcategories of words/expressions that can influence the recognition of this condition by the SAMU MD. This study encourages future investigations on the theme to analyze the quality of medical regulation of emergency calls, particularly for CA. Combinations of key words/expressions could be used to implement protocols to enhance the early recognition of CA by the MD. Also, our results suggest the need to improve communication processes between laypersons and MDs to increase the likelihood of an accurate recognition and consequently early performance of CPR in CA. There are already automatic speech recognition systems that showed better performance in identifying CA than MDs.^25^ Therefore, the combination of words used by the caller, automatically recognized by dedicated systems and machine learning frameworks, could raise the possibility of a large-scale response to emergency calls.

### Limitations

The present study was a pilot of an analysis model that still has several aspects to be adjusted and improved. Among the study limitations, missing or inconsistent registries and the poor quality of recordings and storage of some calls may have caused the loss of cases in the initial sample. In addition, we did not evaluate knowledge or experience level of MDs, or their compliance with regulatory protocols. Likely, we must consider differences in education and knowledge of call handlers and callers. Finally, there is no record of the interval between the occurrence of the event and the call to the emergency services.

In addition, contextual limitations should be considered including the pre-hospital care provided in the Brazilian public system; since it does not make use of effective emergency dispatch protocols or of automatic recognition of the calls received, information may strongly rely on the analysis made by each MD. In this regard, another limitation related to communication may be the regional linguistic variation, and the translation of words/expressions may not reflect cultural and educational diversity of each region.

## Conclusions

The words and expressions considered in the CPS and GS categories and in the “breathing difficulty”, “verbal unresponsiveness”, “facial coloration” and “felling unwell” were identified as confounding factors for the recognition of CA by the MD. On the other hand, the subcategories “not breathing”, “special position”, and “cardiovascular emergencies” were identified as facilitating factors for CA recognition. Understanding these confounders for the recognition of a CA may help in the development of care protocols and more effective training strategies for CA, thereby enhancing communication with laypeople. In contrast, facilitating expressions could be easily incorporated to the protocols for the management of suspected CA. However, further studies in this area are needed due to the multitude of factors that influence the communication process between laypeople and the MD in CA in the out-of-hospital setting.
